# Politics and Health at the WHO Regional Office for South East Asia: The Case of Portuguese India, 1949–61

**DOI:** 10.1017/mdh.2017.34

**Published:** 2017-07

**Authors:** Monica Saavedra

**Affiliations:** Centre for Global Health Histories, Department of History, Berrick Saul Building BS/120, University of York,Heslington, York YO10 5DD, UK

**Keywords:** Portuguese India, Portugal, India, Regional Office for South East Asia, Health, Politics

## Abstract

This paper analyses how the 1950–61 conflict between Portugal and India over the territories that constituted Portuguese India (Goa, Daman and Diu) informed Portugal’s relations with the World Health Organization’s Regional Office for South East Asia (SEARO). The ‘Goa question’ determined the way international health policies were actually put into place locally and the meaning with which they were invested. This case study thus reveals the political production of SEARO as a dynamic space for disputes and negotiations between nation-states in decolonising Asia. In this context, health often came second in the face of contrasting nationalistic projects, both colonial and post-colonial.

## 1 Introduction

Between the end of the Second World War and the forcible end of Portuguese sovereignty over Goa, Daman and Diu – the territories that constituted Portuguese India – in December 1961, Portugal ratified the World Health Organization’s (WHO) Constitution and had a seat at its Regional Office for South East Asia (SEARO), which was based in the Indian capital of New Delhi.^1^ The involvement with SEARO would help Portuguese India foster its health care system, but was also supposed to help the Portuguese government stand up to newly independent post-colonial India’s claims to Goa. SEARO membership was one of a range of diplomatic tools intended to reinforce Portugal’s self-representation as an intercontinental nation-state (*nação pluricontinental*) within the context of a decolonising region (South East Asia) and in direct confrontation with a post-colonial nation state (India).^2^

Drawing on official documents from the Portuguese Overseas and Foreign Affairs Ministries, as well as on published materials accessed at libraries in Lisbon, this article analyses Portugal and Portuguese India’s relations with SEARO, offering an insight into the political disputes and agendas shaping the foundation, constitution, orientation and membership of this regional office in the aftermath of the Second World War and the beginning of decolonisation. It follows the analysis of the politics and diplomacy involved in the establishment and functioning of the WHO and its regional offices, presented in interpretations by Robert Cox and Harold K. Jacobson, Javed Siddiqi, Kelley Lee and Nitsan Chorev, for example.^3^ These consider politics at the WHO from the perspective of the institution’s universalistic principles and globalising objectives. They discuss how politics interfered negatively with the WHO project, placing barriers to and delaying its execution; when and how politics played a part in international health policies in a constructive way; and how the WHO adapted in order to defend its principles in the face of external demands.^4^ The present article will instead consider politics at SEARO from the nation-state perspective. It will look at the different meanings attributed to the regional office, as well as the possibilities envisioned for it, by its member states – Portugal, in this case. In addition, it suggests that from both a national and a local standpoint, success in an international health context may not have been primarily perceived according to how effectively each country conformed to internationally established health administration and delivery practices, but may instead have been measured by how well their involvement with international health institutions served their own national political agenda.

Focusing on the tense relationship between Portugal and India over the ‘Goa question’, attention is drawn to some of the political transactions that were at the roots of SEARO’s formation, determining its scope, practices and effectiveness. It is further suggested that this regional office was yet another of the many shapes taken by Asian regionalism in the period after the Second World War. SEARO is thus revealed not so much as ridden or constrained by politics, but as a product and locus of regional politics. This constitutes one of the ‘multiple forces acting simultaneously to reshape public health policy’, conditioning ‘the ways programs are implemented in particular contexts’,^5^ alongside national and local political interests and multilevel administrative networks.^6^

This article thus looks at ‘supposedly global representations and practices’, exemplified in SEARO’s principles, in a specific setting.^7^ In doing so, it presents this regional office as a ‘contact zone’ – a concept forged by Mary Louise Pratt to refer to ‘social spaces where disparate cultures meet, clash, and grapple with each other, often in highly asymmetrical relations of domination and subordination’.^8^ Expanding on this concept, Gilbert M. Joseph notes that these ‘are not geographic places with stable significations’ but rather ‘simultaneously sites of multivocality, of negotiation, borrowing, and exchange; and of redeployment and reversal’.^9^ Although used here in a rather loose interpretation, this concept summarises the product of negotiations and shifting combinations of national, colonial, post-colonial, regional and international interests that defined SEARO and shaped it and its members’ health policies and practices, which often put politics ahead of health.^10^

## 2 Portugal and the ‘Goa Question’

The Indian army entered Goa in December 1961, 450 years after it came under Portuguese sovereignty. Almost five centuries of uneven European influence and political power resulted in a socially and culturally differentiated society which, in 1956, the Portuguese geographer Orlando Ribeiro described as fundamentally divided between Christians and Hindus, separated by caste and class and where very few, especially ‘among the humble people’, could speak and understand Portuguese.^11^ It was a society deeply influenced by intense emigration that linked the Portuguese and British colonies in Africa and Asia for many decades, and by the shared opened borders with the neighbouring territory of independent post-colonial India.

Despite the fact that by the 1900s Portuguese India as a whole had long lost ‘its strategic, economic, or cultural importance in the empire’,^12^ the political environment (within Goa, in Asia and internationally) after the Second World War rekindled the Portuguese government’s interest. António de Oliveira Salazar, the head of the Portuguese government that in 1933 officially established a dictatorial regime known as the *Estado Novo* (New State), was cognisant of the emerging international views on colonialism. However, Salazar’s government was well aware that giving up Goa would be the first step towards the end of Portugal’s sovereignty over its African colonies (Angola, Cape Verde, Guinea, Mozambique, and Sao Tome and Principe).^13^ Salazar therefore started preparing for the international dispute over Goa that he expected would follow Indian independence. In fact, Goa and the need to make it part of India had first figured in speeches given by Jawaharlal Nehru, India’s first Prime Minister, during his days as Vice-President of the Interim Government of India in 1946. In the light of Nehru’s words, Salazar warned his Minister of the Colonies, Marcelo Caetano:

Attached is a Bombay telegram about Nehru’s declarations about our India. We should start facing up to the problem and gathering all sorts of elements – historical, legal, statistical – to defend ourselves at any international level or even in the eyes of the world.^14^

In 1950, India ‘formally claimed sovereignty over Goa, Daman and Diu’ and tried to negotiate this with the Portuguese government.^15^ However, the latter’s response to India’s claims and to decolonisation in Asia was ‘to alter, not the system, but its legal and institutional foundations’.^16^ The Portuguese Constitution was consequently revised in 1951, in such a way as to make the colonies ‘overseas provinces’ that were deemed integral and inalienable parts of Portugal.^17^ It became an intercontinental nation state (*nação pluricontinental*) and no longer possessed ‘colonies or “non-autonomous territories” as defined by the UN’.^18^ In fact, this revision only emphasised and supposedly provided legal grounds for the affirmation of the unity of Portuguese territory, which had been evoked at the World Health Assembly in 1949 in order to sustain Portugal’s views on the rights of Associate Members of the regional offices.^19^ However, this diplomatic strategy was never effective or convincing. Although Portugal was not the only remaining colonial power in the 1950s, especially where the African continent was concerned, the ‘Goa question’ was an altogether different matter when seen in the context of Asia in general, and independent post-colonial India and its internal and international political agenda in particular.^20^ This ‘question’ came to involve many international actors (including the United Kingdom, the USA and the Vatican), as it unfolded at the United Nations and its agencies as well as at the North Atlantic Treaty Organization (NATO, of which Portugal became a member in 1949).^21^

At this juncture, Portugal’s membership of SEARO was not a case of health planning and governance, but rather a matter of international health diplomacy, with health coming second to the affirmation of sovereignty and nationhood. Taking advantage of the establishment of SEARO and of the rights the WHO ascribed to its member states, Portugal endeavoured to reaffirm its sovereignty over Portuguese India and claim territorial borders in Asia by means of an international organisation which, in principle, aimed to transcend national political interests. That Portugal’s support for the establishment of SEARO and the location of its headquarters in New Delhi should have been sought by India is yet another example of irony and ambiguity that underscores the complexity of health diplomacy as an integral part of the WHO and its regional offices.

## 3 Regionalism in Asia: Situating SEARO

The dominant view of the establishment of the WHO regional offices is that it resulted from the need to incorporate ‘existing regional health organizations, and the creation of new regional bodies to ensure a geographical balance’.^22^ According to Kelley Lee, regionalisation was envisaged from the initial planning stages of the creation of the WHO, as a way of enabling ‘effective institutional links to all member states’.^23^ It became prominent in the early works of the Interim Commission, which was established in 1946 (and was charged with preparing the formation of the WHO) due to objections from members of the Pan-American Sanitary Bureau to the latter’s proposed extinction in favour of the new Organization.^24^ Following this opposition, the Technical Preparatory Committee ‘submitted alternative proposals, the first of which suggested the maintenance of autonomy for regional agencies with a view to transforming them gradually into regional offices under the Organization …’.^25^ The PAHO example is considered a cornerstone of regionalisation, but also an instance of the national and regional interests which marked the discussions on the model to be adopted in order to bring it into effect.^26^

However, little has been written on how India and China took the opportunity created by the need to speedily integrate existing regional organisations into the WHO, and introduced the discussions on the arrangements for the establishment of the regional offices during the debate on the Interim Commission’s report, at the First Health Assembly.^27^ As a result, in January 1949 SEARO became the first regional office to be formally established. Besides being a materialisation of the regionalisation process contemplated in the WHO Constitution, SEARO can be seen in the context of post-1945 Asianism, which was perceived as an alternative to Western influence and power. What is more, according to Sunil Amrith, it also followed European and Asian imperial representations of Asia’s health and disease specificities, as well as their variations within the continent. These perspectives voiced by the European, American and Asian imperial powers intensified the formation of transnational medical and health networks.^28^ SEARO reveals the political dynamics that characterised the, to use Amrith’s term, ‘late-colonial-into-post-colonial’ Asia that reinvented colonial health networks and definitions in accordance with the new political order heralded by decolonisation.^29^ On the other hand, it also added to the subdivisions of the continent and the different configurations they assumed after the Second World War, of which the Asian Relations Conference in New Delhi in March 1947, the Conference on Indonesia in 1949, also in New Delhi, and the 1955 Bandung Conference of the Non-Aligned Movement were significant examples.^30^

SEARO was an ideational and a social construct.^31^ It was based on the universalistic, co-operative and supposedly apolitical principles that marked the WHO Constitution. At the same time, it emerged from the decolonising process and mirrored the notion, discussed at the 1947 Asian Relations Conference, that poverty and underdevelopment, and the illnesses they entailed were the common problem shared by Asian countries.^32^ In addition, it was marked by the political divides within Asia, namely the partition of India and Pakistan and the configurations of the continent as ‘a region where, Nehru thought, India’s new status [independence] should endow it with some kind of leadership’.^33^ Although it was not based on an openly anti-colonial ethos, SEARO incorporated decolonisation effects and tensions. Nevertheless, despite Portugal being, so to speak, a colonial interloper among the rising independent Asian nations that initially formed this regional office, its support was sought and it was accepted as a member. At SEARO, colonial and anti-colonial interests co-existed in a mix of dispute and collaboration.

## 4 Portuguese Politics at SEARO

In line with its drive to strengthen its political power in Asia, in 1948 India initiated contacts in order to secure agreement for its plan to install the Regional Office for South East Asia in New Delhi. Despite the impending tensions with Portugal over Goa, before the First World Health Assembly that took place in June and July 1948, the Indian government contacted the Portuguese Ministry of Foreign Affairs, asking for support. Portugal backed the Indian proposition:

[T]he Government of India would like to create a regional ‘Bureau’ of the World Health Organization in its country and asks for the Portuguese Government’s support for the proposal that will be presented during the first session of that Organization’s Assembly.

The Portuguese Government … has imparted to His Excellency the Minister of Foreign Affairs of this country [India] that instructions will be conveyed to our Geneva delegation telling them to give their approval to the above-mentioned proposal.^34^

A Portuguese delegate was part of ‘the working party for the South East Asia area that met on 2 July 1948’ to discuss ‘the definition of the geographical area’, together with ‘the question of priority with regard to the establishment of a regional organisation’. This working party ‘unanimously agreed’ on the establishment of SEARO ‘with India as its headquarters’.^35^ By supporting India’s proposal and attending the meeting to define SEARO’s composition, Portugal both recognised India’s power and endeavoured to challenge it, by getting directly involved with the regional office and thus reinforcing its claim to sovereignty over Portuguese India. However, only Afghanistan, Burma (Myanmar), Ceylon (Sri Lanka), India and Siam (Thailand) were chosen to be members of the newly established regional office. There were issues regarding membership of the regional offices to be discussed in Geneva before Portugal could actually join under its own terms and in accordance with its aspirations. It thus did not take part in the WHO South East Asia Conference in New Delhi in October 1948, at which the foundations for SEARO were decided (its effective creation depended on the permission of the WHO Executive Board and was formalised in January 1949). During the conference Lieutenant Colonel Chandra Mani, the Deputy Director-General of India’s Health Services, was named Director of SEARO and was confirmed in this post by the Executive Board.^36^ India was decidedly taking the lead in the formation and running of this regional office.

Just as for the other countries in the region, SEARO may perhaps have been able to compensate for the lack of state investment in health in Goa. Years later, when India had already taken over Goa, a member of the Portuguese Ministry for Foreign Affairs acknowledged that ‘the most important aspect of [Portugal’s] collaboration with the WHO Regional Office for South East Asia established in New Delhi was linked to that organisation’s “Operational Plans”, in addition to other sanitary matters’.^37^ However, the official records suggest that it was also a convenient way for Portugal to assert its sovereignty over Portuguese India and display its ‘Portuguese-ness’ in the part of the world where it was most contested. Hence, to the Portuguese government, SEARO membership had a symbolic value: it was a political statement. This much is suggested by the discussions on the rights and obligations of ‘Associate Members and Other Territories’ in the regional offices at the second World Health Assembly (WHA) in 1949. Portugal insisted that ‘States which possessed territories in that region (…), constituted an integral part of the nation and were subject to the same political constitution’ should be considered ‘Member states’. The Counsellor of the Portuguese Embassy to the Holy See, A. R. Pereira, who took part in the second WHA, insisted that Portugal’s overseas territories should be Member States of the regional offices corresponding to the geographical area in which they were located. He based this position on the 1933 Portuguese Constitution, which:

… laid down in Article 1 that the territory of Portugal included: (1) in Europe, Continental Portugal and the Archipelagos of the Azores and Madeira; (2) in West Africa, Cape Verde Islands, Guinea, São Tomé and Principe and their dependencies, S. João Baptista de Gyuda (*sic*), Cabinda and Angola; (3) in East Africa, Mozambique; (4) in Asia, Portuguese India, Macao and their dependencies; (5) in Oceania, Timor and dependencies.^38^

Pereira also noted that ‘the WHO was not qualified to introduce any modification whatsoever in the political status of the Member States’.^39^ From SEARO’s inception, Portugal’s membership was thus fraught with political intents, disputes and calculations, unfolding at both the WHO headquarters and regional office meetings. Regional offices were contact zones for diverse powers and interests. They were neither colonial, nor post-colonial; they illustrate how, in a decolonising world after the Second World War, models, knowledge and techniques co-produced within the worldwide networks created over centuries by European colonialism were invested with new meanings, countering the relationships of power that had long underpinned them.^40^

When SEARO ‘recommended, during its …meeting in New Delhi, that the heads of the health services of the countries belonging to that region meet in Ceylon next September [1950], in order to carry out preliminary studies on common problems’, the Portuguese Overseas Ministry concluded that: ‘Considering the State of India’s [Portuguese India’s] position in South East Asia and the present political situation in that region, it seems to us advantageous for Portugal to accept the above-mentioned recommendation’.^41^ The official report on this meeting of the Regional Committee shows that it ‘was attended by representatives of the six countries in the region, as well as by those from France and Portugal, which, being responsible for the international relations of territories in the region, participate as Members in the Regional Committee’.^42^

The Portuguese delegates to SEARO meetings were mostly Goan doctors – either the Director of the Goa Medical School, or health-service doctors specialising in the topics of the conferences and meetings convened by the regional office. The Overseas and Foreign Affairs Ministries made it clear that they should always be ‘Goans of renowned “Portuguese-ism” ’, prepared to answer the ‘sneaky questions’ asked by Indian journalists.^43^ This became particularly important from 1954 onwards, when political tension between Portugal and India escalated after the latter entered the Portuguese-administered Dadra and Nagar-Avely enclaves in Gujarat (July 1954).^44^ Whenever possible, delegates were also selected from among the few doctors who had received SEARO fellowships for their specialisations. It was important to make the most of their proximity to the international institution’s culture and show that the expertise provided by WHO grants was valued and put to good use by the government. It did not follow that the representatives from Goa saw themselves as mediators of Portugal’s interests, or at least did not just consider themselves mediators or claimants to Portuguese membership rights at SEARO. A random official report from the Portuguese legation in Jakarta, dated August 1960, illustrates the ambiguous situations in which Goa’s representatives at SEARO were placed. It describes the cordial relations of these delegates with their peers at the regional office meeting in Bandung, regardless of nationality and including their Indian colleagues. The Portuguese legation took the opportunity to politically capitalise the delegates’ professional networking by organising a dinner for a few selected guests. The purpose was ‘to affirm here the idea that, in every aspect and unequivocally, a delegation from our India is a delegation from Portugal and should be acknowledged as such’.^45^

Under the authoritarian Portuguese regime’s tight control and in accordance with the intercontinental nation concept, relations between Portuguese India and the Regional Office were managed through an administrative network comprising the Goan government, the Overseas Ministry and the Ministry of Foreign Affairs. This created a chain of command involving institutions diverse in power, place and function, and actors whose interests and meanings could hardly be expected to converge. Control by the Ministries of Overseas and Foreign Affairs also had the dual purpose of ‘reinforcing the national unity principle’ that Portugal endeavoured to demonstrate at every ‘international organisation’, and selecting the information imparted to these organisations.^46^ This is why in 1959 the Overseas Ministry rejected attempts by the Governor of Portuguese India, Manuel António Vassalo e Silva, to be allowed to respond directly to SEARO inquiries in order to simplify communications. The Ministry clearly stated that some information on health and medical assistance might entail ‘political implications that made it necessary to alter them’ or ‘not pass them on at all’.^47^

## 5 Concealing and Unveiling: The Delicate Balance of Politics and Health

The intensification of the political dispute between India and Portugal further complicated the management of Portugal’s interactions at SEARO. It was difficult to maintain the balance between the national interests regarding the ‘overseas provinces’ (especially where they depended on international approval), Goa’s need for support for public health improvements, and active Portuguese membership of SEARO. This quandary is illustrated by a letter dated April 1954, from the Overseas Minister, Manuel Sarmento Rodrigues, to the Governor-General of Portuguese India, Paulo Bénard Guedes, in which he asks whether it would be convenient to invite SEARO to hold its eighth Regional Committee meeting in Goa in 1955. The Governor-General thought it imprudent, replying that:

In the cities of this state there is still no sanitation of the kind required by any population centre, especially a city. The lack of hygiene and other comforts needed to host the more than 40 experienced WHO technical specialists and personnel is therefore obvious. Moreover, the hospitals also display an urgent need for indispensable modernisation. Under these circumstances, I do not find it convenient to choose the city of Goa for the WHO 8th Regional Session scheduled for 1955.^48^

Propaganda was needed and holding a SEARO meeting would be quite impressive, but shortcomings could be used against the Portuguese authorities. There was as much to hide as there was to be displayed. This seems to have been the subtext of the Governor-General’s reply. Although health systems and their material conditions could not by themselves depose regimes, as part of a broader social and political context they might reveal weaknesses and neglect. Given that health issues had become part of the nationalist ideology in Asia, colonial governments could be accused of disregarding the health care of their overseas territories; just as they had been accused of using intrusive and violent public health measures to control epidemics and populations in the past.^49^

Still more expressive of the political calculations behind Portugal’s involvement with SEARO is the succession of changing responses by the Portuguese government to the Regional Office’s invitations. In June 1954 SEARO invited its members – Portugal included (sent to the Minister of Foreign Affairs) – to contribute to an exhibition that was being organised in connection with the seventh session of the SEARO Committee meeting, in September.^50^ The Ministry of Foreign Affairs and the Overseas Ministry both agreed it would be important to accept. Accordingly, in early July 1954 Governor-General Bénard Guedes was asked to prepare a contribution to the exhibition. It was emphasised that this would be fundamental in order to ‘show the advancements achieved in sanitary matters and the interest that our authorities devote to this issue’. Furthermore, it was hoped that Portuguese representation might ‘bring prestige to our administration’ and also ‘mark, once again, the fact that this state [Portuguese India] constitutes a differentiated socio-political entity’.^51^

However, in the wake of the events at Dadra and Nagar-Aveli, everything seemed to change. The Overseas Deputy Secretary and the Minister of Foreign Affairs considered it ‘inopportune’ to send a Goan delegate to the SEARO Committee meeting that year.^52^ Meanwhile, in a display of bureaucratic muddling, contradictory decisions were circulating, and the Overseas Minister finally instructed the government of Portuguese India to nominate the Portuguese delegates to the seventh SEARO committee meeting.^53^ Perhaps not unintentionally, the nominees were one Hindu doctor (Pondorinata Borcar) and one Christian doctor (Aires Rómulo Noronha) doctor.^54^ Both were former WHO fellows (in 1951 and 1950, respectively).^55^ At the same time, the fact that they belonged to the two larger religious communities in Goa suited the Portuguese government’s discourses on the multicultural and multiracial nation.

The materials sent from Goa for exhibition during the meeting nearly missed the event. According to the Portuguese Legation in New Delhi, the Indian authorities were to blame for the ‘abnormal delay of 15 days for a parcel to come from Goa to New Delhi’, and were thus ‘responsible for having reprehensively interfered with the normal duties of the Indian Government as a host-government to an international meeting’.^56^

It was nevertheless in Portugal’s best interest to conduct health diplomacy in a fashion calculated to maintain a flimsy balance between international approval and self-interest. The Portuguese authorities therefore wisely decided not to let their diplomatic relations with India interfere too obviously with Portugal’s SEARO membership and activities. Consequently, in December 1954 the central government, the government of Portuguese India and the latter’s Health Services ungrudgingly acceded to an All-India Medical Conference request to borrow the materials sent to New Delhi and display them during its meeting in Lucknow.^57^ At any rate, it was easier to convey a favourable image of Goa’s health system and improvements through selected photographs and charts than by exposing them to direct observation by inviting SEARO to meet in Portuguese India.

The loan of the materials (probably largely due to the prompt acquiescence of the Director of Goa’s Health Services, Lorindo dos Santos Garcia) did not prevent concurrent backstage political manoeuvrings. The next goal was to ensure that the eighth SEARO committee meeting was not held in New Delhi. As mentioned earlier, the Governor-General of Portuguese India had already opined against holding it in Goa, so given ‘the present phase of Portugal’s relations with India’, the only strategy left was to instruct the Portuguese delegation to ‘support the choice of anywhere other than New Delhi’.^58^

The same caution informed the carefully measured acceptance of foreign aid demonstrated in 1959, when Arne Barkhuus, the Danish ‘director of the [SEARO] health services’, visited Goa. His mission was to ‘familiarise the bureau with [Goa’s] public health problems’ and inform future SEARO assistance to Portuguese India.^59^ Barkhuus’s visit involved discussions between the Overseas and Foreign Affairs Ministries. In line with the European colonial powers’ cautious management of international interventions in their overseas territories, any potential official foreign visit to the Portuguese ‘provinces’ was thoroughly appraised, lest political effects might ensue.^60^ The visit was eventually considered unavoidable and even convenient ‘from a political perspective’, as it ‘might perhaps consolidate [Portugal’s] position as a full member of that Regional Office [SEARO] at a time when some Asian countries have been opposing the presence and membership in those organisations of countries with their metropole in Europe’.^61^

Barkhuus offered internships for Goan doctors who might be interested in specialising in anaesthesia and psychiatry. In view of the lack of proper hygienists at the head of the sanitary control services in Goa, he also proposed fellowships for sanitarian courses to be taught at Indian higher education establishments. Governor-General Bénard Guedes wrote to Lisbon to say that this proposal ‘did not please’ the Government of Goa ‘for several reasons; and if it is only possible for the training to take place in India, we would prefer that instead of the fellowships, an experienced professional be sent here to instruct our personnel’. Better still, ‘if the sanitarian courses could be held outside India, [the Government of Goa] agree[d] that it would be best to have the fellowships for our personnel, rather than having a foreign professional come here’.^62^

This kind of concern to hide or downplay deficiencies was a defensive strategy. It was also symptomatic of the Portuguese authorities’ awareness that, as José Filipe Mesquita (former WHO fellow, health officer and manager of the anti-filariasis campaign in Goa) put it, although better than that of the ‘backward countries’, the ‘sanitary situation of Portuguese India’ could not compare favourably to ‘that of the nations at the forefront of Civilisation’.^63^ In a tense political context, it was thought that these shortcomings might contribute to further criticisms of the Portuguese administration, although there is no evidence they actually did. Nevertheless, the Portuguese government saw health issues as part of the overall political environment and the international management of the ‘Goa question’. At any rate, Portugal’s position was all the frailer, not so much because it was a colonial state among newly independent countries (the United Kingdom was also a member of SEARO), but because its position at SEARO resulted from its claims to a territory that India regarded as its own. Portugal was defying a country that, as Sunil Amrith puts it, ‘lay at the centre of the “new Asia” ’.^64^

## 6 Health Care in Portuguese India: The Local Management of International Health

Encompassed by the ‘Goa question’ that permeated most of the official and public discourse related to every aspect of Goa’s governance, other local factors influenced the cautious management of Portugal’s SEARO membership, as well as the differences between the reality of the existing health system and structure in Portuguese India and what international standards said they should be. What is more, difficulties could arise from the conditions attached to international assistance. Attempts to procure SEARO’s technical help were sometimes checked due to discrepancies between the Regional Office’s means (financial and technical) and expectations, and what the Goan authorities could or would do. In addition, the Regional Office’s shifting guidelines on its technical support were sometimes at odds with the available health structures and medical institutions and the existing health priorities in Goa.

The very fragmentation of Goa’s health care structure and its ethos posed a number of obstacles. It was dispersed between government facilities (hospitals, health centres, sanitary posts, regional infirmaries and dispensaries), charitable and private hospitals and private clinicians. Having said this, in 1958, the Directorate of Health Services (DHS) was the central administrative body, with authority over sixteen local health offices and six sub-offices. Despite a significant dependency on the charitable and private sectors, the DHS clearly made an effort to deliver health care in the rural and remotest areas of Portuguese India, although it is difficult to tell how effective this actually was. Answering either directly to the DHS or to the health offices, there were ‘two regional posts, twenty maternal and child welfare posts and seven welfare posts for mine workers’. In addition, the government ran three general hospitals, a regional infirmary and two smaller ones in rural municipalities, as well as ‘seven free communal clinics’.^65^

Notwithstanding the significant changes in welfare policies in Europe after the Second World War, with a general trend towards universal health and welfare coverage (implied in the principles underlying the WHO Constitution), in Portugal there was resistance to bringing too much responsibility for health care into government hands.^66^ In Goa (as in the metropole) voluntary institutions sustained a local network with social prestige and power that would suffer from increased governmental control over health and welfare services. Goan doctors had their own interests to secure as the providers of qualified manpower in the private, charitable and public health sectors.^67^ Moreover, the prevalent medical culture and health care system in Goa were marked by the training model followed at the Goa Medical School, which offered a generalist medical and surgical curriculum that lacked tuition in most medical specialties and techniques.^68^ Many Goan doctors, especially if they did not pursue their studies elsewhere, were thus a kind of medical ‘jack of all trades’; and family doctors played an important role in people’s perceptions of and demand for health care. In the face of multiple pressures, their loyalties and interests thus remained fragmented.

The SEARO team that visited Goa in 1959 nevertheless acknowledged the drive to integrate all medical and health services under ‘one directing and co-ordinating authority’ controlled by the government.^69^ In fact, 1958 constituted a turning point in Portuguese India’s health services. There was a clear effort to attend to the priority issues established by the WHO, thus accepting a compromise with international health recommendations and guidelines.

Tuberculosis (TB) control and assistance to people infected with the disease, formerly a privately run service, ‘were integrated into the Health Services’ in January 1958.^70^ The same happened to the leprosy hospital, the ‘Mental Hospital’ and the ‘Maternal and Infant Assistance Establishment previously administered and maintained by the Public Assistance Office’ (also a private institution).^71^ The same centralising or at least co-ordinating effort led to the creation of the Overseas Directorate-General of Health and Welfare (a department of the Overseas Ministry) in 1960. The decree that officially established this department said that ‘the multiplicity of official and private bodies that work as welfare instruments in diverse overseas provinces requires a central organisation to unify and coordinate them’.^72^ Some of its centralising functions involved collaboration with international health organisations, as well as proposing overseas representatives to attend international meetings on ‘medicine, hygiene and public health or welfare’.^73^ Unclear in the official sources, however, is the extent to which these reforms were determined by pure foreign policy concerns or by Goan doctors’ interest in making the most of the possibilities offered by SEARO in order to develop their own skills and careers within the process of improving Goa’s health system and structure. In the end, these changes amounted to a mutual accommodation between the increasingly global principles and practices of health administration and health care delivery formalised and disseminated by the WHO and its regional offices, and the locally engendered health system.

This effort was not, however, all that needed to be done in order to respond to Portuguese India’s perceived needs or to what the SEARO advisors deemed necessary for an effective health system. The WHO headquarters and its regional offices urged their member states to adopt international health models that implied a degree of specialisation, well-structured and organised services, and technically qualified staff – elements that were clearly unavailable or not easily attainable in some countries and non-autonomous territories (according to the UN designation), such as Portuguese India. In addition, SEARO increasingly stressed the importance of integrating specialised medical care and control programmes into the general health services.^74^ Indeed, since its outset in 1949, the Regional Office had urged the WHO to ‘concentrate in a practical manner on certain primary public-health requirements’, deeming the ‘lack of certain public-health essentials’ to be major drawbacks for the countries of the South East Asia region.^75^ It was with regard to these aspects that, like most of the countries in SEARO, Portuguese India still fell short of the Regional Office’s goals.

On the other hand, although the WHO and UNICEF technical assistance programmes might help overcome the supposed deficiencies, achieving that kind of organisational level was not regarded as a priority across the board within either colonial or new national governments.^76^ The SEARO Regional Director, Chandra Mani, acknowledged this in the late 1950s, when he wrote about the inadequacy of funds for public health in the countries of the region. He noted that the ‘very limited financial resources of the countries of South East Asia’ made it necessary to use those funds ‘for rapid industrial development’ and ‘the consequent production of more wealth and more purchasing power for the masses’.^77^

In addition, access to delivery of SEARO’s technical assistance was also subject to conditions, as well as being limited by the extent to which internationally established programmes were actually put into practice. For instance, governments within the sphere of operations of the regional offices were expected to provide support to WHO staff working on internationally assisted projects in each country. They were also supposed to continue funding the projects supported by the WHO after the departure of its technical specialists. However, this did not always happen, and the decline of projects implemented with international assistance in the South East Asia region after the international specialists and technicians withdrew was discussed at a number of SEARO meetings.^78^ Furthermore, the limited and varying financial resources of the WHO and its regional offices further complicated their ability to compensate for national governments’ lack of investment in public health.

A few examples drawn from both SEARO reports and accounts by the Portuguese India delegate to SEARO Committee meetings illustrate some of these obstacles to co-operation. The mismatch between local expectations and WHO/SEARO financial constraints and technical procedures explains unsuccessful bids from the Portuguese government for WHO assistance with improving tuberculosis control in Portuguese India. In 1950, the ‘[p]roposal for the provision of Clinical equipment for an Anti-Tuberculosis Dispensary for the Government of Portuguese India’ had been turned down by WHO headquarters, because it ‘could not be included in the 1951 programme for either UNICEF or WHO Regular Budget Assistance due to lack of funds’.^79^ On the other hand, the report of the SEARO Regional Director for 1954–55 (a critical year for diplomatic relations between India and Portugal that may have weighed on health policies) stated that ‘[n]egotiations were started [with Portuguese India] in 1953 for giving WHO assistance in the development of a tuberculosis project. Unfortunately these did not result in a governmental request and the matter has not been reopened since’.^80^

Along similar lines, reporting on his attendance at the 1959 meeting the Goan delegate, José Filipe Mesquita, said he had given up on the idea of asking for WHO support to acquire ‘equipment and materials for mother and child health services and health education’. It had been made clear to him that ‘the WHO only provides equipment and materials to its own projects or government projects set up by managerial staff from the Organization, so that it can have effective control over them’.^81^

In fact, until 1961 the WHO (through SEARO) did not provide any technical support other than the fellowships for disease-dedicated programmes in Portuguese India. The brigades responsible for malaria and filariasis – two diseases designated as priorities within the communicable disease control strategy recommended by both the WHO and SEARO – were part of government-run campaigns and had no support from the regional office.^82^ However, some of the Goan WHO fellows, such as Mesquita, were actually trained to promote those specific programmes locally.

The sparse SEARO support could, nevertheless, be capitalised in the official discourse as an example of Portuguese India’s capabilities, distinctiveness and autonomy. Despite operating solely on local financial resources, the control of (some) communicable diseases was the one area in which Portuguese India stood out. This was acknowledged in the report of the SEARO team that visited Goa in 1959. They particularly noted the ‘extremely valuable results’ attained in some instances, namely the control of malaria and smallpox.^83^ The importance attached to controlling communicable diseases in Portuguese India can be perceived from the local historical and social context. The long-standing notion of the need to protect Goa from diseases coming from India was a decades-old response to historically movable and penetrable borders, but also a statement of Goa’s singularity in relation to India. At the height of the tensions between Portugal and India, in the late 1950s, the image of a disease invasion seemed to herald other forthcoming events.

By talking up the successes of efforts to control some communicable diseases, Lorindo dos Santos Garcia, the Director of the Goa Health Services, vented his patriotic grievances. The marking of the 1957 World Health Day (7 April), which coincided with the celebration of the tenth anniversary of the establishment of the WHO, was an occasion to praise the Organization’s achievements. It was also an opportunity for Garcia to warmly acclaim Goa’s performance in the health field as a dignified way of responding to India’s diplomatic pressure, its economic blockade and its closure of the borders with the Portuguese territory. It further suggests Garcia’s belief that Portuguese India’s health performance would not compromise the Goa government’s legitimacy at SEARO or in relation to the Indian government. Judging from his words, if anything he expected to reinforce that legitimacy. However, his praise was fundamentally directed at the Health Services and its doctors:

Our India’s sanitary condition does not embarrass us. This is due to the silent, permanent and humble, but worthy work of a group of public employees – the Health Officers – and, as their accidental commander, I am very proud. We owe the well-being, security and sanitary peace that we enjoy to them. So commendable and surprising has their efficiency been that the World Organization [*sic*], based in New Delhi, returned the 1956 statistical map to us, calling our attention to it and asking whether there had been some mistake, since it showed only one case of smallpox and none of cholera, plague or typhus. I pleasantly replied that the map was correct; and would also have gladly added that the closure of the borders with the Indian Union had protected us from a more substantial importation.

We benefit from an enviable calm and consider our India an island of thriving freshness in this feverish sea of typhus, cholera, fevers and smallpox that surrounds us. In spite of everything, we are not satisfied; it is necessary to do more and better.^84^

Garcia summarised the tense intersections between national, regional and international in the management and delivery of health in Goa. His discourse illustrates how international health goals, programmes, ways of doing things and assessment mechanisms were locally adjusted and invested with varied uses and meanings. The conflict with India permeated every instance of Portuguese official discourse and informed government decisions. In view of these priorities, by emphasising the role of the Goan doctors this local interpretation of SEARO’s assessment of Portuguese India’s performance according to international health models was a means of affirming Goa’s distinctiveness internationally and engaging with political mobilisation locally. That such successes had been attained despite the lack of any technical support from SEARO reinforced the sense of nationalist self-sufficiency.

## 7 SEARO Support and Last-Minute Diplomacy

Following on from Portugal’s policies after the Second World War, namely the government’s new economic programme, significant reforms were started in Goa. Opening up to international trends and industrialisation marked this period and resulted in the establishment of ‘development plans’. These set the main goals of the Portuguese economic programme and were designed for five-year periods.^85^ The same developmental impetus, also championed by the United Kingdom and France in their colonies, extended to the scientific field.^86^ Accordingly, the Institute of Tropical Medicine in Lisbon, which was the institution that trained Portuguese colonial doctors, undertook a number of scientific missions to the overseas provinces, including Goa.^87^ Portuguese India was also visited by many doctors from the metropole, and for the first time since the late nineteenth century, its Health Services were again headed by European doctors. This may be explained as an attempt on the part of the Portuguese government to regain control over key positions within Goa’s governance structures, especially considering that the authoritative regime faced local political opposition and an increasingly organised pro-India movement, together with pressures from India and uncertain international support.

The official reports for Portuguese India do not specify the amount of money spent separately on health and on education in 1959 and 1960, because both areas came under the same administrative directorate.^88^ The total sum spent on health and education was 233 thousand escudos in 1959 and 2903 million escudos in 1960. This extraordinary increase was mainly due to the building and equipping of hospitals that began in 1960. The investment was nevertheless quite small compared to the amount spent on communications and transport – 24 966 million escudos in 1959 and 10 138 million escudos in 1960.^89^ Still, the central government’s stringent financial administration ‘demanded that in Goa, as in the African colonies, steps be taken towards financial rigour and economic stability’, regardless of their repercussions.^90^ Even so, the public health reforms were apparent in the government of Portuguese India’s response to the WHO world health survey for 1957–60. Each improvement was enumerated, and the involvement of the specialists who had studied with a WHO grant was highlighted, as were the projects that received SEARO support.^91^

In the aftermath of the developmental boost, the Portuguese government requested the WHO’s technical assistance in the nursing and environmental sanitation fields.^92^ Following the WHO-SEARO team’s visit to Goa in 1959, negotiations were started with the Regional Office for the provision of a nursing trainer, supplies and equipment for the eventual establishment of a nursing school. In addition, assistance was agreed for improving the water supply, sewage and waste disposal in a limited area, as well as for training sanitary inspectors.^93^ Subsequent to these negotiations, in September 1960 the government of Goa issued a diploma establishing sanitary agents as a new class of civil servants. Their role was to ‘educate the population on public hygiene and supervise the enforcement of the sanitary laws … concerning environmental sanitation’. The new professional category was to be formed by sanitarians trained on ‘the specialising course that the health services will establish in the city of Goa [Panaji] in collaboration with the World Health Organization’.^94^

SEARO would also assist the Goa Medical School, which ‘needed to strengthen certain departments’.^95^ An ambiguous narrative of glory against all odds, struggle for survival and a number of episodes of imminent closure in the face of stringencies and deficiencies had accompanied the Medical School almost since its establishment.^96^ In 1959, during the visit to the school and its adjacent hospital by Vassalo e Silva (the Governor-General of Portuguese India), João Manuel Pacheco de Figueiredo, the school’s Director, once again called attention to its many needs. He also said that during a visit to Lisbon the previous year he had asked for the Medical School to be included in the overseas development plan, from which it had previously been omitted.^97^ The central and Goan governments’ interests, the Goan doctors’ pleas and SEARO’s privileging of technical training and medical education thus all converged in favour of the ever-depleted Medical School. The will to improve medical teaching and its resources also heralded the change from a generalist medical and surgical teaching and practice to a specialised and technically demanding one; a change that was hotly disputed and would only be completed after Goa’s integration into India.

Nevertheless, when it came to formally agreeing to the kind and form of assistance to be received for the establishment of the nursing course and the environmental sanitation project, the Portuguese government dragged the process through the bureaucratic intricacies of the Foreign Affairs Ministry, the Overseas Ministry and the government of Goa. In addition, it made a point of establishing conditions that would secure the government’s control over the execution of the projects. SEARO sent a first draft of the ‘plan of operations’ for the nursing course on 14 January 1960, asking for an expedite reply. However, the Portuguese Ministry of Foreign Affairs only sent proposed amendments to a few points in the plan in the following January.^98^ These comprised adding a paragraph asserting that:

Experts who are to render advice and assistance to or through the [Portuguese] Government shall be selected by WHO in consultation with the Government. They shall be responsible to WHO. In the performance of their duties, the experts shall act in close consultation with the Government and with persons or bodies so authorised by the Government, and shall comply with instructions from the Government^99^

Moreover, the personnel sent by the WHO were required to speak Portuguese, since English was not widely spoken in Goa. Although SEARO promptly replied favourably to these requests, the agreements between the two parties for both projects were signed as late as November 1961, a month before the Indian army took over Goa.^100^

At about the same time, the government of Portuguese India was using its last diplomatic resources as an active SEARO member. It managed to obtain the Overseas and the Foreign Affairs Ministries’ permission to fulfil the Regional Office’s request for a cultural contribution to its building in New Delhi. Even though the central government in Lisbon made it clear the expense would fall entirely on the Goan budget, the government of Goa commissioned two painted tile panels that were meant to decorate the walls of two rooms at the Regional Office building. In October 1961, the panels arrived in Karachi on their way to New Delhi. At the time, few were aware that these interactions were going to be brought to a sudden end by India’s military takeover of Goa, two months later. The Governor-General of Portuguese India, Vassalo e Silva, wrote hopefully that the ‘requested artistic contribution’ would ‘remain as a cultural marker and a representative of Portugal at the headquarters of the WHO Regional Office for South East Asia’.^101^ It would, in fact, soon become a remnant of a waning colonial power in the South Asian sub-continent.

## 8 Concluding Remarks

Official sources regarding Portugal’s involvement with SEARO during the last years of Portuguese sovereignty over Goa, Daman and Diu reveal a situation in which the Portuguese government used health care and medicine as political and diplomatic instruments. SEARO presented Portugal with a stage on which to enact its self-representation as an indivisible intercontinental nation. From the perspective of the Portuguese authorities, SEARO became an important locus for upholding international claims to Portugal’s boundaries in Asia – especially considering that the country only became a UN member in 1956. That perception, purged of imperialistic discourse and buttressed by a nationalist one, contrasted starkly with the ‘conception of Asia’ envisaged by the countries of the region as they emerged from decolonisation.^102^

Portugal was fighting a losing battle against India, a newly independent nation-state eager to boost its international prestige and build up its influence and power in South Asia. This article suggests that despite the Cold War and the influence of economic and political powers such as the USA and the USSR at WHO headquarters,^103^ other meaningful and influential power disputes were also taking place and reshaping international health models at the regional, national and local levels. Consequently, it shows how SEARO was both product and locus of a regional health diplomacy that often subjugated health to political interest. This is demonstrated as much by the Portuguese government’s motivations in backing India’s request for support for the intention to create a regional office with its headquarters in New Delhi, as by India’s eagerness in taking the lead and making that proposition at the First World Health Assembly. The increasingly globalised structures of health administration, health care delivery and medical education and practice are here looked at from a national and local perspective – and a highly politicised one too – within the context of the Portuguese government’s dispute with India over Goa and of India’s aspirations to a leading position in Asia. At the same time, it highlights the co-production of the global and the national, showing that Portugal’s performance at SEARO was also an acknowledgement that this country’s political agenda – the permanence and cohesion of its intercontinental nation-state – could only be fulfilled by its legitimisation at the international level.^104^ This would, supposedly, be achieved through globalist institutions, namely SEARO, and practices invested with different meanings and intentions, but formed in a context irretrievably shaped by ‘various sorts of colonialism’.^105^ This paper thus corroborates the importance of considering multiple scales and actors when analysing the politics of international, and indeed, global health.

## Footnotes

1.
Throughout this paper Portuguese India and Goa will be used interchangeably, as they were during the period under study. This was mainly due to the fact that Goa was the largest district and where Pangim (Panaji), the capital of Portuguese India, stood.

2.
For the use of the term ‘*nação pluricontinental*’ see, for instance, Cláudia Castelo, ‘*O Modo Português de Estar no Mundo’: O Luso-Tropicalismo e a Ideologia Colonial Portuguesa (1933–61)* (Porto: Afrontamento, 2011), 58; and Fernando Rosas, ‘Goa, ou o princípio do fim’, preface to Orlando Ribeiro, *Goa em 1956 – Relatório ao Governo* (Lisbon: Comissão Nacional para as Comemorações dos Descobrimentos Portugueses, 1999), 11–24.

3.
Nitsan Chorev, *The World Health Organization between North and South* (Ithaca, NY: Cornell University Press, 2012); Kelley Lee, *The World Health Organization (WHO)* (London: Routledge, 2009); Javed Siddiqi, *World Health and World Politics: The World Health Organization and the UN System* (Columbia, SC: University of South Carolina Press, 1995); Robert W. Cox and Harold K. Jacobson, *The Anatomy of Influence: Decision Making in International Organization* (New Haven, CT: Yale University Press, 1973).

4.
Chorev (*op. cit.* (note 3)) uses the concept of ‘strategic adaptation’.

5.
Mark Nichter, *Global Health: Why Cultural Perceptions, Social Representations, and Biopolitics Matter* (Tucson, AZ: University of Arizona Press, 2008), 108.

6.
See Sanjoy Bhattacharya, *Expunging Variola: The Control and Eradication of Smallpox in India, 1947–77* (New Delhi: Orient Longman, 2006).

7.
Warwick Anderson, ‘Introduction: Postcolonial Technoscience’, *Social Studies of Science*, 32, 5–6 (October–December 2002), 643–58: 645.

8.
Mary Louise Pratt, *Imperial Eyes: Travel Writing and Transculturation* (London and New York: Routledge, 2008), 7.

9.
Gilbert M. Joseph, ‘Close encounters: toward a new cultural history of U.S.–Latin American relations’, in Gilbert M. Joseph, Catherine C. Legrand and Ricardo D. Salvatore (eds), *Close Encounters of Empire: Writing the Cultural History of U.S.–Latin American Relations* (Durham, NC: Duke University Press, 1998), 3–46: 5. On the use of the ‘contact zones’ concept, see also Anderson, *op. cit.* (note 7).

10.
On the complexity of the connections between national, colonial, regional and global, analysed in a different context (the global biopolitics and global health), see Alison Bashford, ‘Global Biopolitics and the History of World Health’, *History of the Human Sciences*, 19, 1 (2006), 67–88.

11.
Orlando Ribeiro, *Goa em 1956: Relatório ao Governo* (Lisbon: Comissão Nacional para as Comemorações dos Descobrimentos Portugueses, 1999).

12.

Cristiana Bastos , ‘Doctors for the Empire: The Medical School of Goa and its Narratives’, Identities: Global Studies in Culture and Power, 8, 4 (2010), 517–548: 522.

13.
Maria Manuel Stocker also wrote: ‘Portugal did not have the economic and military means that would permit a conventional war outside the metropole. The possible defence was against subversion and the priorities of the colonial policy were the African colonies. The Portuguese strategic orientation in [Portuguese] India was therefore subject to the exigencies of keeping the rest of the empire’ (Maria Manuel Stocker, *Xeque-Mate a Goa* (Alfragide, Lisbon: Texto, 2011), 334).

14.
António de Oliveira Salazar to the Minister of the Colonies, Marcelo Caetano, cited in Yves Léonard, ‘O Ultramar Português’, in Francisco Bethencourt and Kirti Chaudhuri (eds), *História da Expansão Portuguesa*, Vol. V: Último Império e Recentramento (1930–98) (Lisbon: Círculo de Leitores, 1999), 31–50. Stocker, *op. cit.* (note 13), 81.

15.
António Costa Pinto, ‘Portugal e a resistência à descolonização’, in Francisco Bethencourt and Kirti Chaudhuri (eds), *História da Expansão Portuguesa*, Vol. V: Último Império e Recentramento (1930–98) (Lisbon: Círculo de Leitores, 1999), 51–64: 51, 52.

16.
Valentim Alexandre, ‘O Império Colonial no século XX’, in Valentim Alexandre (ed.), *Velho Brasil Novas Áfricas – Portugal e o Império (1808–1975)* (Porto: Afrontamento, 2000), 195. Also, Castelo, *op. cit.* (note 2).

17.
Lei n.2:048 – Constituição, *Diário do Governo*, I, 117 (11-06-1951).

18.
Léonard, *op. cit.* (note 14), 36.

19.
WHO, Official Records of the World Health Organization No. 21. Second World Health Assembly. Rome,13 June to 2 July 1949. Decisions and Resolutions. Plenary Meetings Verbatim Records. Committees Minutes and Reports. Annexes. Geneva, Palais des Nations, December 1949, 316 (http://apps.who.int/iris/handle/10665/85600).

20.
On European (particularly British and French) colonialism in Africa and the political manoeuvres to postpone decolonisation, see, for instance, Jane Burbank and Frederick Cooper, *Empires in World History: Power and the Politics of Difference* (Princeton, NJ, and Oxford: Princeton University Press, 2010); John Kent, *The Internationalization of Colonialism: Britain, France and Black Africa, 1939–56* (Oxford: Clarendon Press, 1992). On India’s national and international political agenda, see, for example, Taru Dalmia and David M. Malone, ‘Historical Influences on India’s Foreign Policy’, *International Journal*, 67, 4, Canada after 9/11 (Autumn 2012), 1029–49; Sudipta Kaviraj, ‘Modernity and Politics in India’, *Daedalus*, 129, 1, Multiple Modernities (Winter 2000), 137–62; Sugata Bose and Ayesha Jalal (eds), *Nationalism, Democracy and Development: State and Politics in India* (New Delhi: Oxford University Press, 2015 [1997]); Bhikhu Parekh, ‘Nehru and the National Philosophy of India’, *Economic and Political Weekly*, 26, 1–2 (January 5–12 1991), 35–9, 41–3, 45–8.

21.
Stocker, *op. cit.* (note 13); Luís Nuno Rodrigues, ‘Os Estados Unidos e a questão de Goa em 1961’, *Ler História*, 42 (2002), 61–90.

22.
Lee, *op. cit.* (note 3), 30. See also Siddiqi, *op. cit.* (note 3).

23.
Lee, *op. cit.* (note 3), 30.

24.
See, for example, Marcos Cueto and Steven Palmer, *Medicine and Public Health in Latin America: A History* (New York: Cambridge University Press, 2015); Marcos Cueto, *The Value of Health: A History of the Pan-American Health Organization* (Rochester, NY: University of Rochester Press, 2007).

25.
‘WHO’, *International Organization*, 1, 1 (February 1947), 134–36: 134. On the Pan-American Sanitary Bureau (called Pan-American Health Organization, from 1959 onwards) negotiations and agreement with the WHO, and its influence on the regionalisation process, see Cueto and Palmer, *op. cit.* (note 24); Cueto, *op. cit.* (note 24).

26.
Lee, *op. cit.* (note 3); Siddiqi, *op. cit.* (note 3); Harold K. Jacobson, ‘WHO: medicine, regionalism, and managed politics’, in Robert W. Cox and Harold K. Jacobson (eds), *The Anatomy of Influence: Decision Making in International Organizations* (New Haven, CT, and London: Yale University Press, 1973), 175–215.

27.
Charles S. Ascher, ‘Current problems in the World Health Organization’s program’, *International Organization*, 6, 1 (February 1952), 27–50; WHO, Official Records of the World Health Organization, No. 13, First World Health Assembly, Geneva, 24 June to 24 July 1948. Plenary Meetings, Verbatim Records. Main Committees – Minutes and Reports. Summary of Resolutions and Decisions. Palais des Nations, Geneva, December 1948 (particularly pages 32 and 33) (http://apps.who.int/iris/bitstream/10665/85592/1/Official_record13_eng.pdf).

28.
Sunil S. Amrith, ‘The internationalization of health in Southeast Asia’, in Tim Harper and Sunil S. Amrith (eds), *Histories of Health in Southeast Asia: Perspectives on the Long Twentieth Century* (Bloomington, IN, and Indianapolis: Indiana University Press, 2014), 161–79.

29.
Sunil S. Amrith, ‘Health and sovereignty in the New Asia’, in Deepak Kumar and Raj Sekhar Basu (eds), *Medical Encounters in British India* (Oxford: Oxford University Press, 2013), 99.

30.
See Christophe Jaffrelot, ‘India’s Look East Policy: An Asianist Strategy in Perspective’, *India Review*, 2, 2 (2003); Prasenjit Duara, ‘Asia Redux’, *The Journal of Asian Studies*, 69, 4 (November 2010), 963–983; Amitav Acharya, ‘Asia Is Not One’, *The Journal of Asian Studies*, 69, 4 (November 2010), 1001–13.

31.
On the notion of regions as ideational and social constructs, see Acharya, *ibid*.

32.
Amrith, ‘Health and sovereignty’, *op. cit.* (note 29), 105.

33.
Jaffrelot, *op. cit.* (note 30), 42. See also Acharya, *op. cit.* (note 30).

34.
Director-Geral dos Negócios Económicos e Consulares, Ministério dos Negócios Estrangeiros, to Chefe do Gabinete do Ministro das Colónias, 7-06-1948, Arquivo Histórico Ultramarino (AHU), Lisbon, 55 1B MU DGAPC MÇ 1948–58.

35.
WHO, Official Records of the World Health Organization, No. 13, *op. cit.* (note 27), 267.

36.
Office of the United Nations in Geneva, Information Centre, Press Release No. 539, WHO/69 – World Health Organization. WHO Southeast Asia Conference Recommends New Delhi as Headquarters Site for Regional Organization, 5-10-1948, WHO Archive, 1st and 2nd Generation Files, 902-1-2 Public information; WHO, Official Records of the World Health Organization No. 14. Reports of the Executive Board, First and Second Sessions. Held in Geneva from 16 to 28 July and from October to 11 November 1948. Geneva, Palais des Nations, December 1948 (see p. 16) (http://apps.who.int/iris/bitstream/10665/85593/1/Official_record14_eng.pdf).

37.
Silva Nogueira, Ministério dos Negócios Estrangeiros, ‘Informação de Serviço’, 22-01-1962, Arquivo Histórico Diplomático, Ministério dos Negócios Estrangeiros (AHD/MNE), Lisbon, P.O.I. 13, Repartição dos Organismos Políticos Internacionais, ProcN 924, 2300, Bureau Regional para o Sudeste Asiático, Geral 1955–63.

38.
WHO, Official Records of the World Health Organization No. 21, *op. cit.* (note 19), 316.

39.

*Ibid*., 315.

40.
Warwick Anderson and Hans Pols, ‘Scientific Patriotism: Medical Science and National Self-Fashioning in Southeast Asia’, *Comparative Studies in Society and History*, 54, 1 (2012), 93–113; Shamshad Khan, ‘Systems of Medicine and Nationalist Discourse in India: Towards “New Horizons” in Medical Anthropology and History’, *Social Science and Medicine*, 62 (2006), 2786–97; Gyan Prakash, *Another Reason: Science and the Imagination of Modern India* (Princeton, NJ: Princeton University Press, 1999); Frederick Cooper and Randall Packard (eds), *International Development and the Social Sciences: Essays on the History and Politics of Knowledge* (Berkeley, CA, and London: University of California Press, 1997).

41.
Ministério das Colónias, Direcção Geral de Fomento Colonial, Repartição dos Serviços Económicos, 14-11-1949, AHU, Lisbon, 55 1B MU DGAPC MÇ 1948–58.

42.
WHO, Official Records of the World Health Organization No. 30. Work of the WHO, 1950. Annual Report of the Director-General to the World Health Assembly and to the United Nations. Geneva, Palais des Nations, April 1951, 95 (http://apps.who.int/iris/bitstream/10665/85609/1/Official_record30_eng.pdf).

43.
Director-Geral da Administração Política e Civil to Governador-Geral do Estado da Índia, Lisbon, 19-04-1956, AHD/MNE, Lisbon, MU/GM/GNP/RRI/0682/12372; Director-Geral dos Negócios Políticos e da Administração Interna to Director-Geral da Administração Política e Civil, Ministério do Ultramar, Lisbon, 31-03-1956, AHD/MNE, Lisbon, MU/GM/GNP/RRI/0682/12372.

44.
Stocker, *op. cit.* (note 13).

45.
Legação de Portugal em Djakarta [*sic.*] to Ministro dos Negócios Estrangeiros, August 1960, fl. 2. AHD/MNE, Lisbon, P.O.I. 13, Repartição dos Organismos Políticos Internacionais, ProcN 924, 2300, Bureau Regional para o Sudeste Asiático, Geral 1955–63. The cautious selection of Portugal’s representatives at the international organisations was part of the dictatorial regime’s practice of keeping records on anyone holding or applying for a civil service position (implemented by the ‘Polícia International e de Defesa do Estado’ (PIDE, International and State Defence Police]), which was designed to control any ‘subversive activities’ against the regime (see, for example, Fernando Rosas, *Salazar e o Poder: A Arte de Saber Durar* (Lisbon: Tinta da China, 2015), particularly 196–202.

46.
Direcção Geral de Administração Política e Civil, Ministério do Ultramar, to Governo Geral do Estado da Índia, 21-08-1959, AHU, Lisbon, 3213 1B MU DGSA MÇ 1954–63(1).

47.

*Ibid*.

48.
Governador-Geral to Ministro do Ultramar, 21-04-1954, AHD/MNE, Lisbon, MU/GM/GNP/RRI/0682/12372.

49.
See, for example, Samiksha Sehrawat, *Colonial Medical Care in North India: Gender, State, and Society, c.1840–1920* (New Delhi: Oxford University Press, 2013), particularly chapter 2; Anderson and Pols, *op. cit.* (note 40); Pratik Chakrabarti, ‘ “Signs of the times”: Medicine and Nationhood in British India’, *Osiris*, 24, 1, Science and National Identity (2009), 188–211; David Arnold, ‘Colonial Medicine in Transition: Medical Research in India, 1910–47’, *South Asia Research*, 14, 1 (Spring 1994), 10–35.

50.
United Nations, World Health Organization, Regional Office for South East Asia, Ref. PI/740/54, 16 June 1964, to Ministry of Foreign Affairs, Government of Portugal, AHD/MNE, Lisbon, MU/GM/GNP/RRI/0682/12372.

51.
2^a^Secção, Urgente, OMS Bureau Regional para o Sudeste da Ásia, 7^a^ Sessão, to Governador Geral do Estado da Índia, 12 July 1954, AHD/MNE, Lisbon, MU/GM/GNP/RRI/0682/12372.

52.
Urgente, 5/65/316/C/41, OMS, Bureau Regional do Sudueste [*sic.*] da Ásia, to Director Geral dos Negócios Políticos e da Administração Interna, 16-08-1954; Ministério dos Negócios Estrangeiros, Direcção-Geral dos Negócios Políticos e da Administração Interna, Proc. 396,31, n1113, to Director-Geral da Administração Política e Civil, 27-08-1954, AHD/MNE, Lisbon, MU/GM/GNP/RRI/0682/12372.

53.
Ministério do Ultramar, Gabinete do Ministro, n2406, to Direcção Geral de Administração Política e Civil, A. A. Peixoto Correia, chefe do gabinete, 4-09-1954, AHD/MNE, Lisbon, MU/GM/GNP/RRI/0682/12372.

54.
5473 316/C/41, to Director Geral dos Negócios Políticos e da Administração Interna, Ministério dos Negócios Estrangeiros, 15-09-1954, AHD/MNE, Lisbon, MU/GM/GNP/RRI/0682/12372.

55.
Direcção dos Serviços de Fazenda e Contabilidade, Mapa: Província do Estado da Índia. Relação dos funcionários que beneficiaram de bolsa de estudo concedida pela O.M.S. desde 1950, annex to document No. 1013/6920/D/3528, from Governador Geral do Estado da Índia, Manuel Vassalo e Silva to Ministro do Ultramar, 4-08-1960, AHD/MNE, Lisbon, MU/GM/GNP/RRI/0785/12349.

56.
Copy, Legação de Portugal, Nova Delhi, n159, Proc. 16,4, to Ministry of External Affairs, Government of India, 22-09-1954, AHD/MNE, Lisbon, MU/GM/GNP/RRI/0682/12372.

57.
WHO, Regional Office for South East Asia, C. Mani, Regional Director, to Ministry of Foreign Affairs, Government of Portugal (Copy), 9-11-1954; Governo Geral do Estado da Índia, Direcção de Saúde e Higiene, Paulo Bénard Guedes, Governador-Geral do Estado da Índia, to Ministro do Ultramar, 22-12-1954, AHD/MNE, Lisbon, MU/GM/GNP/RRI/0682/12372.

58.
Ministério do Ultramar, Direcção-Geral de Administração Política e Civil, ‘7^a^Sessão do Comité Regional para o Sudeste da Ásia’, Ruy Heitor, 22-09-1954, AHD/MNE, Lisbon, MU/GM/GNP/RRI/0682/12372.

59.
Director-Geral dos Negócios Políticos e da Administração Interna, Ministério dos Negócios Estrangeiros, to Director-Geral de Administração Política e Civil, Ministério do Ultramar, N.726, Proc. 924, 2300, 25-03-1958, AHD/MNE, Lisbon, P.O.I. 13, Repartição dos Organismos Políticos Internacionais, Proc N 924, 2300, Bureau Regional para o Sudeste Asiático, Geral 1955–63.

60.
On the management of and even resistance to international interventions in the European colonial territories, especially in Africa, see Frederick Cooper, ‘Development, Modernization, and the Social Sciences in the era of Decolonization: The Example of British and French Africa’, *Revue d’Histoire des Sciences Humaines*, 10 (2004), 9–38. Writing about the creation of the Combined Commission for the Technical Co-operation in Africa south of the Sahara (CCTA), of which Portugal was a founding member, Kent also points to France’s interest in establishing an Inter-African Medical Committee to mediate contacts with the WHO. The objective was to hinder the announced WHO intention to establish a regional office in Africa, thus ‘preventing other organizations [from] obtaining a foothold in the colonies’ (Kent *op. cit.* (note 20), 269).

61.
Director-Geral dos Negócios Políticos e da Administração Interna, Ministério dos Negócios Estrangeiros, to Director-Geral de Administração Política e Civil, Ministério do Ultramar, N.726, *op. cit.* (note 59).

62.
Governador-Geral to Ministro do Ultramar, N.919/s 1958, AHU, Lisbon, 3213 1B MU DGSA MÇ 1954–63(2).

63.
‘Palestra do Dr. José Filipe Mesquita’, *Revista Farmacêutica*, 1 (January–March 1957), 10. It is worth noticing, however, that health indicators in the metropole were also poor. In 1960 Portugal had one of the highest child mortality rates in Europe – 77.5 per thousand in Portugal compared to 22.5 per thousand in the UK (http://www.pordata.pt/DB/Europa/Ambiente+de+Consulta/Tabela, consulted on 26 January 2016).

64.
Sunil S. Amrith, *Decolonizing International Health: India and Southeast Asia, 1930–65* (Basingstoke: Palgrave Macmillan, 2006), 79.

65.
WHO, First Report on the World Health Situation, Chapter VIII: Countries Reviews, A11/P&B/6 Add. 1, 12 May 1958, 234 (http://www.who.int/iris/handle/10665/109240#sthash.xyP4xqop.dpuf).

66.
On welfare policies after Second World War, see, for instance, Dorothy Porter, *Health, Civilization and the State: A History of Public Health from Ancient to Modern Times* (London and New York: Routledge, 1999); Virginia Berridge, Martin Gorsky and Alex Mold, *Public Health in History* (Maidenhead: Open University Press, 2011). On the principles set out in the WHO Constitution, see Constitution of the World Health Organization (http://apps.who.int/gb/bd/PDF/bd47/EN/constitution-en.pdf?ua=1).

67.
Despite the separation in 1945 of the Goa Medical School from the Health Services (to which it had been linked since its official establishment in 1847), it remained the only source of doctors for Portuguese India’s medical service. Very few European doctors made a career in Goa.

68.
João Manuel Pacheco de Figueiredo, ‘Escola Médico-Cirúrgica de Goa: Esboço histórico’, *Arquivos da Escola Médico-Cirúrgica de Goa*, A, 3 (1960), 119–227: 192–3; Almerindo Lessa, ‘Uma urgente questão nacional: Da necessidade de dignificar a prática e o ensino da medicina na Índia Portuguesa’, offprint of *O Médico* (October 1952), particularly 15–17.

69.
The team defined itself as a WHO team, but its members included Lucien Bernard, Director of the Office of Health Services; Walter C. [last name illegible], Regional Advisor in Environmental Sanitation; and F. Lillywhite, Regional Advisor in Nursing, all from SEARO (World Health Organization, South East Asia Regional Office, Report on visit to Portuguese India, 1959, AHU, Lisbon, 3213 1B MU DGSA MÇ 1954-63(2)).

70.
Lorindo A. dos Santos Garcia, ‘The Health Services in Portuguese India’, offprint of *Anais do Instituto de Medicina Tropical*: Número especial dedicado aos VI Congressos Internacionais de Medicina Tropical e de Paludismo, XV, supplement 2 (September 1958), 301.

71.

*Ibid*, 301.

72.
Ministério do Ultramar, ‘Decreto-Lei n.43 353’, *Legislação do Estado da Índia, 1961*, Vol. 1 (Imprensa Nacional, 1961), 72.

73.

*Ibid.*, 72.

74.
WHO, Regional Office for South East Asia, Eighth Annual Report of the Regional Director to the Regional Committee for South East Asia, July 1955–July 1956, SEA/RC9/2 (http://repository.searo.who.int/bitstream/123456789/5276/59/rdr56_Complete.Pdf).

75.
WHO, Regional Office for S. E. Asia, C. Mani to W. Bonne, Director, Division of Planning, WHO, Geneva, 11-05-1949, WHO Archive, 1st and 2nd Generation Files, SEARO 1949 902-1-8.

76.
See Amrith, *Decolonizing International Health*, *op. cit.* (note 64).

77.
WHO, South East Asia Regional Office,Thirteenth Annual Report of the Regional Director to the Regional Committee for South East Asia, 1 July 1960–1 August 1961 SEA/RC 14/2, vii (http://www.who.int/iris/handle/10665/130740#sthash.5mpTsFFw.dpuf). On Nehru’s economic development strategies and industrialisation planning before and after independence, see Ramachandra Guha, *India After Gandhi: The History of the World’s Largest Democracy* (Basingstoke and Oxford: Pan Books, 2007), 203; David C. Engerman, ‘The Romance of Economic Development and New Histories of the Cold War’, *Diplomatic History*, 28, 1 (January 2004), 23–54; Parekh, *op. cit.* (note 20).

78.
Teófilo de Jesus Fernandes and Joaquim Cabral, Relatório dos trabalhos da Delegação Portuguesa à 10^a^Reunião do Comité Regional do Sudeste da Ásia da O.M.S., Goa, 1-10-1957, AHU, 3213 1B MU DGSA MÇ 1952–63(1); José Filipe Mesquita, Serviço da República, Direcção Provincial dos Serviços de Instrução e Saúde do Estado da India, Relatório, 30-10-1959, AHU, 3213 1B MU DGSA MÇ 1952–63(1); WHO, Regional Office for South East Asia, Sixth Annual Report of the Regional Director of the Regional Committee for South East Asia, July 1953–July 1954, SEA/RC7/2, New Delhi, August 6, 1954, v (http://apps.who.int/iris/handle/10665/131014); WHO, Report of the Eleventh Session of WHO Regional Committee for South East Asia, New Delhi, 24–30 September 1958, SEA/RC11/24, 13 (http://repository.searo.who.int/bitstream/123456789/5279/56/rdr59_Complete.Pdf).

79.
WHO, Regional Office for South East Asia, SEA/REPORT/50-12, Monthly Progress Report, December 1950. To the Director General, World Health Organization, Geneva, from the Regional Director, Regional Office for S. E. Asia, New Delhi, WHO Archive, 1st and 2nd Generation Files, CC 7/8.

80.
WHO, Regional Office for South East Asia, Seventh Annual Report of the Regional Director to the Regional Committee for South East Asia, July 1954–July 1955, 110 (http://repository.searo.who.int/bitstream/123456789/5275/57/rdr55_Complete.Pdf).

81.
Mesquita, *op. cit.* (note 78), fl. 2.

82.
WHO, Eleventh World Health Assembly, First Report on the World Health Situation, A11/P&B/6, 15-04-1958 (http://www.who.int/iris/handle/10665/109240); World Health Organization, Fifteenth World Health Assembly, Second Report on the World Health Situation (Part I), A15/P&B/3 Part I, 13-04-1962 (http://www.who.int/iris/handle/10665/135733#sthash.Uy4NuM2Z.dpuf); WHO, Regional Office for South East Asia, Eighth Annual Report of the Regional Director to the Regional Committee for South East Asia, July 1955–July 1956, SEA/RC9/2 (http://repository.searo.who.int/bitstream/123456789/5276/59/rdr56_Complete.Pdf); WHO, Regional Office for South East Asia, Ninth Annual Report of the Regional Director to the Regional Committee For South East Asia, July 1956–July 1957, SEA/RC10/2 (http://repository.searo.who.int/bitstream/123456789/5277/58/rdr57_Complete.Pdf); WHO, Regional Office for South East Asia, Twelfth Annual Report of the Regional Director, 1959 August–June 1960, SEA/RC13/2 (http://repository.searo.who.int/bitstream/123456789/5280/59/rdr60_Complete.Pdf).

83.
WHO, SEARO, Report on visit to Portuguese India, 1959, fl. 6, 7, AHU, Lisbon, 3213 1B MU DGSA MÇ 1954–63(2).

84.
Lorindo dos Santos Garcia, cited in ‘Em comemoração do 10Aniversário da Organização Mundial de Saúde’, *Revista Farmacêutica*, 1 (January–March 1957), 4.

85.
On the development plans, see, for instance, João L. César das Neves, ‘O crescimento económico português no pós-guerra: Um quadro global’, *Análise Social*, 29, 128, 4(1994), 1005–34.

86.
Cláudia Castelo, ‘Investigação científica e política colonial portuguesa: Evolução e articulações, 1936–74’, *História, Ciências, Saúde – Manguinhos*, 19, 2 (April–June 2012), 391–408; Frederick Cooper and Randall Packard, introduction in Frederick Cooper and Randall Packard (eds), *International Development and the Social Sciences: Essays on the History and Politics of Knowledge* (Berkeley, CA, Los Angeles and London: University of California Press, 1997).

87.
Figueiredo, *op. cit.* (note 68), particularly 204-7. ‘Missão à Índia de professores da metrópole’, *Arquivos da Escola Médico-Cirúrgica de Goa*, A, 27 (1954), 1–31; F.J.C. Cambournac, ‘Sobre o combate ao sezonismo no território de Goa (Estado da Índia)’, *Anais do Instituto de Medicina Tropical*, VI (December 1949), 7–40.14771459

88.
Garcia, *op. cit.* (note 70).

89.
Portugal, Instituto Nacional de Estatística, *Anuário Estatístico do Ultramar*, 1960, 193 (http://inenetw02.ine.pt:8080/biblioteca/search.do).

90.
Stocker, *op. cit.* (note 13), 66.

91.
Repartição dos Serviços de Saúde do Estado da India, Resposta ao questionário sobre a situação sanitária do mundo, AHU, Lisbon, 3210 1B MU DGSA MÇ. 1957–60.

92.
WHO, South East Asia Regional Office, *op. cit.* (note 69).

93.
WHO, South East Asia Regional Office, *op. cit.* (note 69); Plan of Operation for the Provision of Technical Assistance by the World Health Organization under the Expanded Programme of Technical Assistance to the Government of Portugal for Portuguese India, J. Hall Themido, Government of Portugal, 15-11-1961, C. Mani, Regional Director, World Health Organization, 17-10-1961; Plan of Operation for Environmental Sanitation Project, Portuguese India 8, J. Hall Themido, Government of Portugal, 14-11-1961, C. Mani, Regional Director, World Health Organization, 21-11-1961; AHD/MNE, Lisbon, P.O.I. 13, Repartição dos Organismos Políticos Internacionais, Proc N 924, 2300, Bureau Regional para o Sudeste Asiático, Geral 1955–63.

94.
Governo-Geral, Diploma Legislativo n.2020, *Legislação do Estado da Índia, 1960*, vol. II (Imprensa Nacional, 1961), 279–80: 280.

95.
WHO, Regional Office for South East Asia, Proposed Programme and Budget Estimates for 1961, New Delhi, July 1959 (http://repository.searo.who.int/bitstream/123456789/17724/1/sea_rc12_3.Pdf).

96.
See Cristiana Bastos, ‘O ensino da medicina na Índia colonial portuguesa: Fundação e primeiras décadas da Escola Médico-cirúrgica de Nova Goa’, *História, Ciências, Saúde – Manguinhos*, 11, supl. 1 (2004), 11–39; Cristiana Bastos, ‘Medicina, império e processos locais em Goa, século XIX’, *Análise Social*, 42, 182 (2007), 99–122; Bastos, *op. cit.* (note 12).10.1590/s0104-5970200400040000215446241

97.
‘A deplorável situação do Hospital Escolar de Goa’, *Jornal do Médico* (15 August 1959), 824.

98.
Director General for Political Affairs, A. Franco Nogueira, to the Regional Director for S.E. Asia, World Health Organization, Lisbon, 9-01-1961. AHD/MNE, Lisbon, P.O.I. 13, Repartição dos Organismos Políticos Internacionais, ProcN 924, 2300, Bureau Regional para o Sudeste Asiático, Geral 1955–63.

99.
WHO, Regional Office for South East Asia, Regional Director, C. Mani, to the Ministry of Foreign Affairs, Government of Portugal, 27-01-1961, AHD/MNE, Lisbon, P.O.I. 13, Repartição dos Organismos Políticos Internacionais, ProcN 924, 2300, Bureau Regional para o Sudeste Asiático, Geral 1955–63.

100.
Plan of Operation for the Provision of Technical Assistance …; Plan of Operation for Environmental Sanitation Project, *op. cit.* (note 93). The signed documents do not mention the agreement about Portuguese-speaking WHO personnel; but the Regional Director, C. Mani, had written to the Portuguese Ministry of Foreign Affairs, asking the Portuguese government to ‘kindly accept this letter as assurance’ that the SEARO would ‘make every effort to recruit staff members with sufficient working knowledge of Portuguese’. Mani further explained that ‘It will be embarrassing to put this matter in the actual agreement’, *ibid*.

101.
Manuel António Vassalo e Silva, Governor-General of Portuguese India, cited in Joaquim Ferreira da Silva, Director-Geral da Direcção-Geral de Saúde e Assistência do Ultramar, 12-04-1961, AHU, Lisbon, 3213 1B MU DGSA MÇ 1952-63(1).

102.
See Amrith, *op. cit.* (note 64), 76–7.

103.
Marcos Cueto, *Cold War Deadly Fevers: Malaria Eradication in Mexico, 1955–75* (Washington DC: Woodrow Wilson Center, 2007).

104.
The same is suggested by Bhattacharya (*op. cit.* (note 6)) when he analyses how the Indian government emphasised its role and success in the smallpox eradication programme, giving it a nationalist meaning while endeavouring to affirm its regional leadership and attain international recognition.

105.

Warwick Anderson , ‘Making Global Health History: The Postcolonial Worldliness of Biomedicine’, Social History of Medicine, 27, 2 (2014), 372–384: 381.

